# ROS-Induced DNA Damage Enhances Sensitivity to PARP Inhibition in HSC3 and SCC25 Head and Neck Squamous Cell Carcinoma Cell Lines

**DOI:** 10.3390/cimb48070692

**Published:** 2026-07-05

**Authors:** Negar Taghavi Pourianazar

**Affiliations:** Medical Laboratory Techniques, Vocational School of Health Services, Istanbul Aydin University, Istanbul 34295, Turkey; negarpourianazar@aydin.edu.tr

**Keywords:** PARP inhibitors, olaparib, reactive oxygen species, DNA damage, head and neck squamous cell carcinoma, synergistic effect

## Abstract

Background: Head and neck squamous cell carcinoma (HNSCC) remains a highly aggressive malignancy with poor clinical outcomes. Although poly(ADP-ribose) polymerase (PARP) inhibitors have shown promising activity in tumors with homologous recombination deficiency, their efficacy in BRCA wild-type HNSCC remains limited. Reactive oxygen species (ROS)-induced DNA damage may increase cellular dependence on DNA repair pathways and thereby enhance sensitivity to PARP inhibition. This study investigated whether ROS-mediated DNA damage could sensitize BRCA wild-type HNSCC cells to the PARP inhibitor olaparib. Methods: BRCA wild-type HSC-3 and SCC-25 HNSCC cell lines were exposed to H_2_O_2_ to induce oxidative stress. Intracellular ROS levels were quantified using DCFDA assays, DNA double-strand breaks were evaluated by γ-H2AX ELISA, PARP activity was assessed by ELISA, and cell viability was determined using MTT assays. Expression levels of DNA repair genes (PARP1, PARP2, BRCA1, BRCA2, RAD51, and MLH1), checkpoint kinases (ATM, ATR, and CHK1), the homologous recombination regulator FANCD2, and redox defense genes (NQO1, GPX4, and SLC7A11) were analyzed by qRT-PCR. Therapeutic selectivity was assessed using HGF-1 normal human gingival fibroblasts as a normal cell control. Apoptosis was measured through caspase-3/7 activity assays, and drug interactions were evaluated using the Chou–Talalay method. Results: H_2_O_2_ treatment increased intracellular ROS levels in both cell lines, accompanied by significant induction of DNA damage as demonstrated by elevated γ-H2AX levels. ROS induction markedly enhanced olaparib sensitivity, significantly reducing IC_50_ values in both HSC-3 and SCC-25 cells. Combined H_2_O_2_ and olaparib treatment produced strong synergistic cytotoxicity, suppressed DNA repair, checkpoint kinase, and redox defense gene expression, and increased caspase-3/7 activity compared with control cells. Importantly, the combination demonstrated selective cytotoxicity toward cancer cells, with normal HGF-1 cells retaining significantly higher viability. Conclusions: ROS-induced DNA damage significantly enhances the anti-tumor activity of olaparib in BRCA wild-type HNSCC cells through a functional synthetic lethal-like interaction involving the simultaneous collapse of DNA repair capacity, checkpoint activation, and oxidative stress buffering, culminating in apoptosis induction. These findings support the rationale for combining ROS-generating therapies with PARP inhibitors in HNSCC treatment.

## 1. Introduction

Head and neck squamous cell carcinoma (HNSCC) represents a significant global health burden, accounting for approximately 3–5% of cancer-related deaths worldwide. Annually, more than 890,000 new HNSCC cases are diagnosed, with over 450,000 deaths reported [1,2]. HNSCC is typically diagnosed at advanced stages, with 5-year survival rates below 50% [2]. Conventional treatment modalities such as surgery, radiotherapy, and chemotherapy show limited efficacy and cause severe adverse effects, necessitating the development of novel and effective therapeutic strategies [3,4].

DNA repair mechanisms are critical for maintaining cellular genomic integrity [5]. Poly(ADP-ribose) polymerase (PARP) family enzymes play crucial roles in DNA damage response [6]. PARP1 and PARP2 are responsible for repairing single-strand breaks (SSBs) and certain double-strand breaks (DSBs) [7]. Upon DNA damage detection, PARP enzymes utilize NAD^+^ substrate to generate poly(ADP-ribose) (PAR) chains, which facilitate recruitment of DNA repair proteins to damage sites [8].

PARP inhibitors block PARP enzymatic activity, thereby inhibiting DNA repair mechanisms [9]. BRCA1 or BRCA2 mutant cells (defective homologous recombination repair pathway) are highly sensitive to PARP inhibitors, a functional synthetic lethal-like interaction [10,11]. However, BRCA wild-type cells (functional homologous recombination pathway) show relative resistance to PARP inhibitors [12]. Since most HNSCC cases are BRCA wild-type, PARP inhibitors demonstrate limited single-agent efficacy [13]. Therefore, strategies to enhance PARP inhibitor sensitivity in BRCA wild-type HNSCC are critically needed [14].

Reactive oxygen species (ROS) are highly reactive molecules generated through metabolic activities, mitochondrial respiration, and exogenous stress factors (radiotherapy, chemotherapy) [15]. ROS can cause oxidative damage to DNA, proteins, and lipids [16]. ROS-induced DNA damage can generate DSBs and SSBs, triggering cellular DNA repair mechanisms [17]. If DNA damage cannot be repaired, cells undergo apoptosis [18]. This property is exploited in cancer therapy, where radiotherapy and chemotherapy partially kill cancer cells through ROS generation [19].

The phosphorylation of histone H2AX at serine 139 (γ-H2AX) is one of the earliest and most sensitive markers of DNA double-strand breaks [20]. γ-H2AX has been extensively used as a biomarker for DNA damage induced by various stress factors, including ionizing radiation and chemotherapy [21]. The accumulation of γ-H2AX at DNA damage sites triggers recruitment of DNA repair proteins and activates checkpoint mechanisms [22].

Recent studies have demonstrated that combining DNA-damaging agents with PARP inhibitors produces synergistic cytotoxicity in various cancer types [23,24]. For example, topotecan combined with PARP inhibitors showed enhanced cytotoxicity in ovarian cancer cells [25]. Similarly, radiotherapy combined with PARP inhibitors demonstrated improved efficacy in prostate cancer models [26]. However, the specific mechanisms underlying this synergy in HNSCC remain poorly understood [27].

This study hypothesizes that ROS-induced DNA damage may sensitize BRCA wild-type HNSCC cells to PARP inhibitors. By inducing multiple DNA lesions, ROS places cells under “DNA repair burden.” Under these conditions, PARP inhibition may impair BER/SSB repair, forcing cells into apoptosis [28,29].

**Figure 1:**
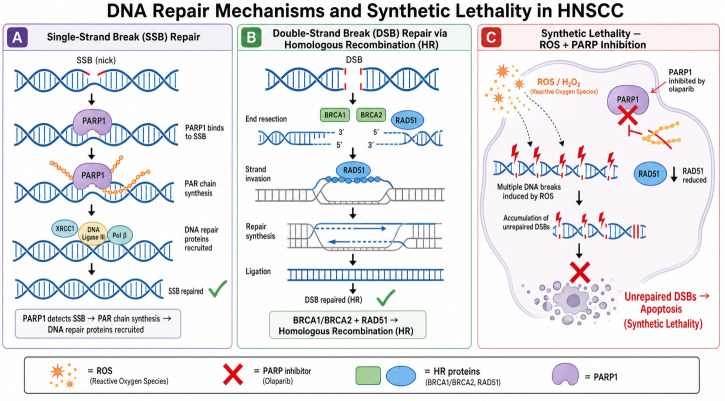
DNA Repair Mechanisms and Synthetic Lethality in HNSCC. (**A**) Single-Strand Break (SSB) Repair: Upon detection of a single-strand break (SSB, indicated by a red nick on the DNA helix), PARP1 (purple oval) binds to the damage site and synthesizes poly(ADP-ribose) (PAR) chains (orange beads), which recruit downstream repair proteins including XRCC1, DNA Polymerase β (Pol β), and DNA Ligase III to complete SSB repair. (**B**) Double-Strand Break (DSB) Repair via Homolo-gous Recombination (HR): Upon detection of a double-strand break (DSB, indicated by a complete break in the DNA he-lix), BRCA1 and BRCA2 (green rectangles) coordinate with RAD51 (blue oval) to perform end resection, strand invasion, repair synthesis, and ligation, restoring genomic integrity through the HR pathway. (**C**) Synthetic Lethality—ROS + PARP Inhibition: Exogenous reactive oxygen species (ROS, represented by orange spark symbols).

Accordingly, the potential role of ROS-induced DNA damage in enhancing PARP inhibitor (olaparib) sensitivity was investigated in HSC-3 and SCC-25 HNSCC cell lines. These findings may demonstrate therapeutic potential for combining ROS-inducing therapies with PARP inhibitors in HNSCC treatment [30]. The proposed mechanism involves the disruption of both single-strand break (SSB) and double-strand break (DSB) repair pathways, leading to a functional synthetic lethal-like interaction (Figure 1).

## 2. Materials and Methods

### 2.1. Cell Culture and Reagents

Human HNSCC cell lines HSC-3 (derived from tongue squamous cell carcinoma) and SCC-25 (derived from buccal mucosa squamous cell carcinoma) were obtained from Prof. Dr. Ömer Faruk Bayrak, Department of Medical Genetics, Faculty of Medicine, Yeditepe University. Cells were cultured in Dulbecco’s Modified Eagle Medium (DMEM) supplemented with 10% fetal bovine serum (FBS) and 1% penicillin/streptomycin. Cells were maintained at 37 °C in a humidified 5% CO_2_ incubator and passaged every 3–4 days using 0.25% trypsin-EDTA. The PARP inhibitor olaparib and hydrogen peroxide (H_2_O_2_) were obtained from commercial sources. N-acetylcysteine (NAC), a ROS scavenger, was used as a control for ROS-specific effects. Olaparib was dissolved in DMSO and diluted in culture medium before treatment. Final DMSO concentration did not exceed 0.1%.

To assess therapeutic selectivity, human gingival fibroblasts (HGF-1, obtained from Prof. Dr. Ömer Faruk Bayrak) were used as a normal cell control. HGF-1 cells originate from the oral cavity and represent a biologically relevant normal cell model for comparison with the HNSCC cell lines. HGF-1 cells were cultured in Dulbecco’s Modified Eagle Medium (DMEM; Gibco, Grand Island, NY, USA) supplemented with 10% FBS and 1% penicillin/streptomycin under the same conditions as the cancer cell lines.

### 2.2. Treatment Protocol

HSC3 and SCC25 cells were subjected to various treatments depending on the experimental group. Untreated cells maintained in normal culture medium served as the negative control to establish baseline levels for all assays. For ROS induction, cells were treated with 50 µM or 100 µM H_2_O_2_ for 24 h, acting as an inducer condition. In antioxidant rescue experiments, cells were pretreated with 5 mM N-acetylcysteine (NAC) for 2 h, followed by 100 µM H_2_O_2_ treatment for 24 h. For PARP inhibition, cells were treated with 1 µM or 5 µM olaparib for 24–48 h. For combination treatments, cells were pretreated with 100 µM H_2_O_2_ for 24 h, followed by 5 µM olaparib treatment for 24–48 h. HGF-1 normal cells were subjected to identical treatment protocols as the cancer cell lines to evaluate differential cytotoxic responses.

### 2.3. Intracellular ROS Level Measurement (DCFDA Assay)

Oxidative stress was induced by treating HSC-3 and SCC-25 cells (5 × 10^4^ cells/well, 96-well black plate) with H_2_O_2_ at concentrations of 50 µM and 100 µM for 24 h. For antioxidant rescue experiments, cells were pretreated with 5 mM NAC for 2 h prior to H_2_O_2_ exposure (100 µM, 24 h). Intracellular ROS levels were quantified using 2′,7′-dichlorofluorescin diacetate (DCFDA, Invitrogen, Eugene, OR, USA), a non-fluorescent probe that becomes fluorescent upon oxidation by ROS [31]. After treatment, cells were washed with HBSS and incubated with 10 µM DCFDA at 37 °C for 30 min in the dark. Fluorescence intensity was measured using a microplate reader (Varioskan™ LUX Multimode; Thermo Fisher Scientific, Waltham, MA, USA) (excitation: 485 nm, emission: 535 nm).

ROS levels were expressed as a percentage of the control group (100%). Fold-change was calculated as: (Mean fluorescence of treated cells/Mean fluorescence of control cells) × 100. Results are presented as mean ± SD of three independent replicates.

### 2.4. DNA Double-Strand Breaks Measurement (γ-H2AX ELISA)

DNA double-strand breaks (DSBs) were measured using phosphorylated histone H2AX (γ-H2AX) ELISA kit (Abcam, Cambridge, UK) [32]. γ-H2AX is a well-established marker of DNA double-strand breaks, with phosphorylation at serine 139 occurring within minutes of DSB formation. HSC-3 and SCC-25 cells (2 × 10^5^ cells/well, 96-well plate) were subjected to the following treatments: (1) Control; (2) H_2_O_2_ treatment (50 µM H_2_O_2_ for 24 h); (3) H_2_O_2_ high dose (100 µM H_2_O_2_ for 24 h); (4) NAC pretreatment (5 mM for 2 h, followed by 100 µM H_2_O_2_ for 24 h). After treatment, cells were fixed with 4% paraformaldehyde (PFA) in PBS for 15 min at room temperature to preserve cellular morphology and protein structure. Cells were then permeabilized with 0.1% Triton X-100 in PBS for 10 min to allow antibody penetration into the nucleus. Non-specific binding sites were blocked with 5% bovine serum albumin (BSA) in PBS for 1 h at room temperature.

Cells were incubated with anti-phospho-histone H2AX (Ser139) monoclonal antibody (1:1000 dilution in 1% BSA/PBS, Cell Signaling Technology, Danvers, MA, USA) at 4 °C overnight to allow specific binding to phosphorylated H2AX at DSB sites. Following primary antibody incubation, cells were washed three times with PBS-Tween (0.05% Tween-20 in PBS) to remove unbound antibody. HRP-conjugated goat anti-mouse secondary antibody (1:5000 dilution in 1% BSA/PBS) was applied for 1 h at room temperature. After three additional washes with PBS-Tween, TMB substrate (3,3′,5,5′-tetramethylbenzidine) was added and incubated for 30 min at room temperature in the dark to allow color development. The enzymatic reaction was stopped by adding 2 M H_2_SO_4_ (100 µL per well). Optical density (OD) was measured at 450 nm using a microplate reader (ThermoFischer, Waltham, MA, USA, Varioskan™ LUX Multimode). Results were expressed as a percentage of the control group (untreated cells = 100%), with fold-change calculated as:

γ-H2AX fold-change = (Mean OD of treated cells/Mean OD of control cells) × 100.

Data are presented as mean ± standard deviation (SD) of three independent biological replicates.

### 2.5. PARP Activity Assay

PARP enzymatic activity was measured using a PAR accumulation ELISA kit (Trevigen, Gaithersburg, MD, USA) [33]. HSC3 and SCC25 cells (2 × 10^5^ cells/well, 96-well plate) were subjected to the following treatments: (1) Control; (2) H_2_O_2_ (100 µM, 24 h) to induce DNA damage and activate PARP; (3) Olaparib (1 µM, 24 h) for partial PARP inhibition; (4) Olaparib (5 µM, 24 h) for maximal PARP inhibition; (5) H_2_O_2_ + Olaparib combination (100 µM H_2_O_2_ for 24 h followed by 5 µM olaparib for 24 h). After treatment, cells were fixed with 4% PFA for 15 min, permeabilized with 0.1% Triton X-100 for 10 min, and blocked with 5% BSA for 1 h. Cells were incubated with anti-poly(ADP-ribose) antibody (1:1000, 4 °C overnight), washed three times, and incubated with HRP-conjugated secondary antibody (1:5000, 1 h). TMB substrate was added for 30 min, the reaction was stopped with 2 M H_2_SO_4_, and OD was measured at 450 nm.

### 2.6. Cell Viability Assay

Cell viability was assessed using the 3-(4,5-dimethylthiazol-2-yl)-2,5-diphenyltetrazolium bromide (MTT) assay [34]. HSC-3 and SCC-25 cells were seeded in 96-well plates and treated with various concentrations of olaparib (0–10 µM) in the presence or absence of H_2_O_2_ (100 µM). Following 48 h of treatment, MTT reagent was added, and cells were incubated for 4 h at 37 °C. The formazan precipitate was dissolved in DMSO, and absorbance was measured at 570 nm. Cell viability was calculated as a percentage of untreated control cells. Cell viability was assessed at 24 h and 48 h post-treatment, depending on the experimental groups.

Cell viability was measured using the 3-(4,5-dimethylthiazol-2-yl)-2,5-diphenyltetrazolium bromide (MTT) assay. HSC-3 and SCC-25 cells (5 × 10^3^ cells/well, 96-well plate) were subjected to control, H_2_O_2_ (100 µM), olaparib (0–10 µM), or combination treatment. After 48 h treatment, 20 µL MTT solution (5 mg/mL in PBS) was added to each well and incubated for 4 h at 37 °C. Formazan crystals were dissolved in 200 µL DMSO. OD was measured at 570 nm (reference: 650 nm). Cell viability was calculated as:

Cell Viability (%) = (OD_treatment/OD_control) × 100.

Dose–response curves were generated using non-linear regression analysis (variable slope, four-parameter logistic model) in GraphPad Prism 8.0, and IC_50_ values were calculated.

### 2.7. Quantitative Real-Time PCR (qRT-PCR)

Total RNA was isolated from HSC-3 and SCC-25 cells using TRIzol reagent (Invitrogen) [35]. RNA concentration and purity were determined by NanoDrop spectrophotometer (A260/A280 ratio > 1.8). cDNA was synthesized using a reverse transcription kit (Applied Biosystems). qRT-PCR was performed using SYBR Green master mix (Applied Biosystems) and real-time PCR system (Applied Biosystems 7500; Applied Biosystems, Foster City, CA, USA). The expression levels of DNA repair genes (PARP1, PARP2, BRCA1, BRCA2, RAD51, MLH1), DNA damage checkpoint kinases (ATM, ATR, CHK1), homologous recombination regulator (FANCD2), and redox defense genes (NQO1, GPX4, SLC7A11) were evaluated. GAPDH was used as an internal control. Relative gene expression was calculated using the 2^ΔΔCT^ method.

### 2.8. Apoptosis Assessment (ELISA-Based Caspase Assay)

Apoptosis was measured using a caspase-3/7 activity ELISA kit (Abcam, Cambridge, UK) [36]. Caspase-3/7 activation is a hallmark of apoptosis and serves as a quantitative marker of programmed cell death. HSC3 and SCC25 cells (2 × 10^5^ cells/well, 96-well plate) were subjected to the following treatments: (1) Control; (2) H_2_O_2_ (100 µM for 24 h) to induce ROS-mediated apoptosis; (3) Olaparib (5 µM for 24 h) for PARP inhibition-induced apoptosis; (4) H_2_O_2_ + Olaparib combination (100 µM H_2_O_2_ for 24 h followed by 5 µM olaparib for 24 h) to assess synergistic apoptosis induction. After treatment, cells were lysed using the lysis buffer provided in the kit for 10 min at room temperature. Cell lysates were transferred to a 96-well ELISA plate and processed according to the kit protocol. HRP-conjugated caspase-3/7 substrate was added and incubated for 1 h at room temperature. TMB substrate was added for color development (30 min), the reaction was stopped with 2 M H_2_SO_4_, and OD was measured at 450 nm.

Caspase-3/7 activity was expressed as a percentage of the control group (100%). Fold-change was calculated as: (Mean OD of treated cells/Mean OD of control cells) × 100.

### 2.9. Synergistic Effect Analysis (Chou–Talalay Method)

Synergy was quantified using the Chou–Talalay method [37]. This method calculates the combination index (CI) based on the median-effect principle. The CI is calculated as:

CI = (D)_1_/(Dx)_1_ + (D)_2_/(Dx)_2_

where (D)_1_ and (D)_2_ are the concentrations of drug 1 and drug 2 used in combination and (Dx)_1_ and (Dx)_2_ are the concentrations of drug 1 and drug 2 alone that produce the same effect.

The dose reduction index (DRI) was also calculated: DRI = (Dx)_m_/(D)_m_, where (Dx)_m_ is the dose of drug m that produces a given effect as a single agent, and (D)_m_ is the dose of drug m in the combination that produces the same effect.

Interpretation of CI values: CI < 0.9 (Synergistic interaction), 0.9 ≤ CI ≤ 1.1 (Additive interaction), CI > 1.1 (Antagonistic interaction).

### 2.10. Statistical Analysis

All experiments were performed in three independent biological replicates, and data are presented as mean ± standard deviation (SD). Statistical significance was determined using two-way ANOVA followed by Tukey’s post hoc test to account for the two independent variables (treatment condition and cell line). For qRT-PCR analysis, statistical significance testing was performed on the normally distributed ΔCt values prior to calculating the relative fold-change (2^−ΔΔCT^) for graphical representation. Dose–response curves were generated using non-linear regression analysis (curve fit: [Inhibitor] vs. normalized response—Variable slope) to calculate IC_50_ values and their 95% confidence intervals. Synergistic effects were assessed using the Chou–Talalay combination index method, where CI < 0.9 indicates synergy. All statistical analyses were performed using GraphPad Prism 8.0 (GraphPad Software, San Diego, CA, USA). A *p*-value < 0.05 was considered statistically significant.

## 3. Results

### 3.1. H_2_O_2_ Treatment Induces ROS Production in HSC-3 and SCC-25Cells

**Figure 2:**
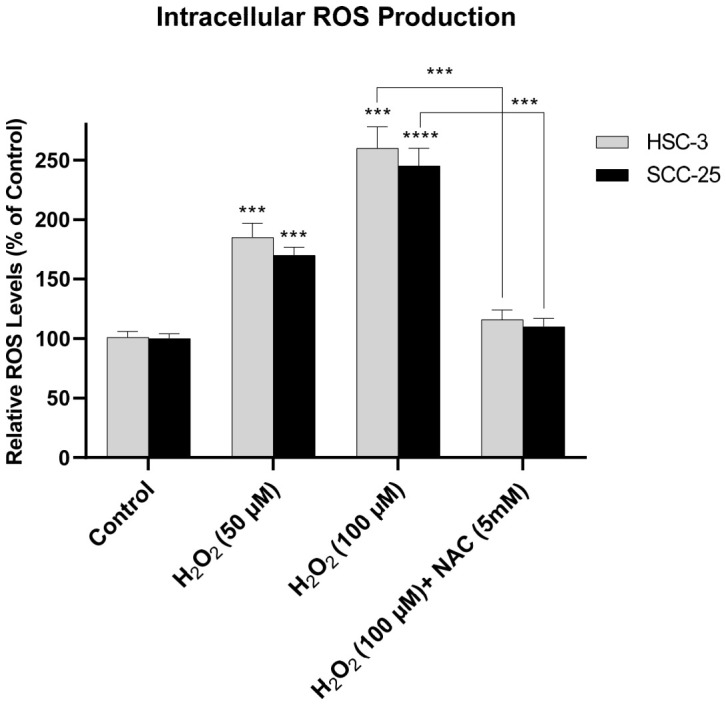
Intracellular ROS Levels. H_2_O_2_ induces dose-dependent ROS production in HSC-3 and SCC-25 cells. Cells were treated with H_2_O_2_ (50 or 100 µM, 24 h) or pretreated with NAC (5 mM, 2 h) followed by H_2_O_2_ (100 µM, 24 h). ROS levels were measured by DCFDA assay and expressed as a percentage of the control (100%). H_2_O_2_ at 100 µM increased ROS to 260% ± 18% (HSC-3) and 245% ± 15% (SCC-25) (*p* < 0.001). NAC pretreatment reduced ROS to 115% ± 8% (HSC-3) and 110% ± 7% (SCC-25) (*p* < 0.001). Data are presented as mean ± SD (n = 3). Statistical significance: *** *p* < 0.001, **** *p* < 0.0001 compared to control.

To establish the baseline ROS-inducing capacity of H_2_O_2_, we treated HSC-3 and SCC-25 cells with increasing concentrations of H_2_O_2_ for 24 h and measured intracellular ROS levels using the DCFDA assay, which detects ROS through fluorescence intensity proportional to ROS concentration. H_2_O_2_ treatment at 50 µM increased ROS levels to 185% ± 12% in HSC-3 cells and 170% ± 10% in SCC-25cells, representing 1.85-fold and 1.70-fold increases compared to control, respectively. At the higher concentration of 100 µM, H_2_O_2_ treatment induced more robust ROS production, increasing ROS levels to 260% ± 18% in HSC-3 cells and 245% ± 15% in SCC-25cells, representing 2.6-fold and 2.45-fold increases compared to control, respectively. To confirm that ROS production was responsible for the observed effects, we pretreated cells with 5 mM N-acetylcysteine (NAC), a well-established antioxidant, for 2 h prior to H_2_O_2_ (100 µM) exposure for 24 h. NAC pretreatment significantly reduced H_2_O_2_-induced ROS increase, with ROS levels reduced to 115% ± 8% in HSC-3 cells and 110% ± 7% in SCC-25 cells, representing 73% and 76% reductions in H_2_O_2_-induced ROS, respectively (Figure 2). These findings demonstrate that H_2_O_2_ induces dose-dependent ROS production in both HNSCC cell lines and that this ROS production can be effectively reversed by antioxidant treatment, confirming the specificity of the ROS-inducing effect.

### 3.2. ROS-Induced DNA Double-Strand Breaks

**Figure 3:**
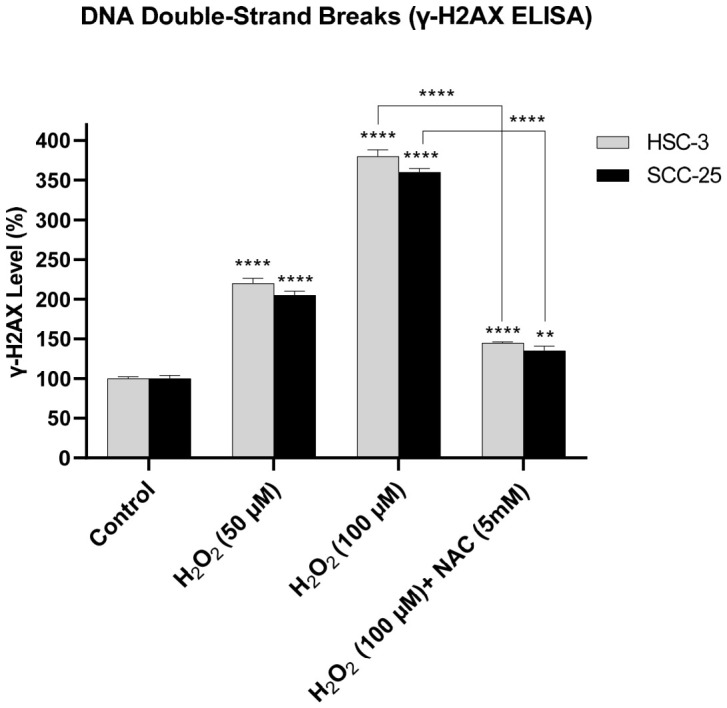
DNA Double-Strand Breaks. ROS induces DNA double-strand breaks measured by γ-H2AX ELISA. Cells were treated with H_2_O_2_ (50 or 100 µM, 24 h) or pretreated with NAC (5 mM, 2 h) followed by H_2_O_2_ (100 µM, 24 h). γ-H2AX levels at 100 µM H_2_O_2_ increased to 380% ± 28% (HSC-3) and 360% ± 25% (SCC-25). NAC pretreatment reduced γ-H2AX to 145% ± 12% (HSC-3) and 135% ± 10% (SCC-25). Pearson correlation analysis showed a strong positive correlation between ROS and γ-H2AX levels (HSC-3: r = 0.94; SCC-25: r = 0.96). Data are mean ± SD of three independent replicates. Statistical significance: ** *p* < 0.01 and **** *p* < 0.0001 compared to control.

To investigate whether ROS-induced oxidative stress translates into DNA damage, we measured DNA DSBs using γ-H2AX ELISA, which quantifies phosphorylation of histone H2AX at serine 139—a hallmark marker of DSBs that accumulates at DNA damage sites within minutes of break formation. H_2_O_2_ treatment at 50 µM increased γ-H2AX levels to 220% ± 18% in HSC-3 cells and 205% ± 15% in SCC-25cells, representing 2.2-fold and 2.05-fold increases compared to control, respectively. At 100 µM, H_2_O_2_ treatment induced more substantial DNA damage, with γ-H2AX levels increasing to 380% ± 28% in HSC-3 cells and 360% ± 25% in SCC-25cells, representing 3.8-fold and 3.6-fold increases compared to control, respectively. NAC pretreatment significantly reduced H_2_O_2_-induced DNA damage, with γ-H2AX levels reduced to 145% ± 12% in HSC-3 cells and 135% ± 10% in SCC-25 cells, representing 62% and 65% reductions in H_2_O_2_-induced DNA damage, respectively. Pearson correlation analysis revealed a strong positive correlation between ROS levels (measured by DCFDA) and γ-H2AX levels (measured by ELISA), with correlation coefficients of r = 0.94 in HSC-3 cells and r = 0.96 in SCC-25cells, indicating very strong positive correlations (Figure 3). These findings demonstrate that ROS is the primary driver of DNA double-strand breaks in HNSCC cells, with a strong quantitative relationship between ROS levels and DSB formation. The strong correlation suggests that ROS-induced DNA damage is a direct and proportional consequence of ROS production.

### 3.3. H_2_O_2_ Treatment Increases PARP Activity

**Figure 4:**
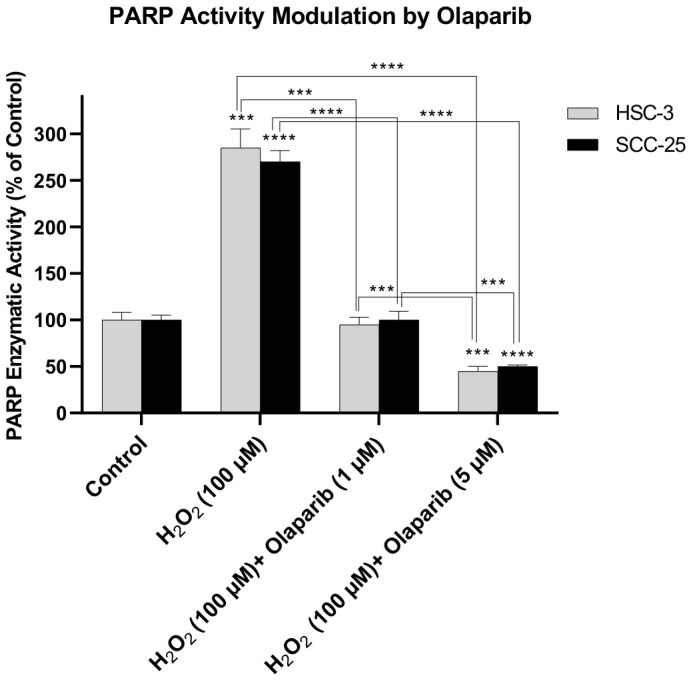
PARP Enzymatic Activity. H_2_O_2_-induced DNA damage activates PARP, which is inhibited by olaparib. Cells were treated with H_2_O_2_ (100 µM, 24 h), olaparib (1 or 5 µM, 24 h), or combinations. H_2_O_2_ increased PARP activity to 285% ± 20% (HSC-3) and 270% ± 18% (SCC-25). Olaparib at 5 µM reduced PARP activity to 45% ± 5% (HSC-3) and 50% ± 4% (SCC-25), demonstrating dose-dependent PARP inhibition. Data are presented as mean ± SD (n = 3). Statistical significance: *** *p* < 0.001 and **** *p* < 0.0001 compared to control.

To determine whether ROS-induced DNA damage triggers activation of DNA repair mechanisms, we measured PARP enzymatic activity using ELISA-based quantification of PAR accumulation. PAR chains are synthesized by PARP enzymes in response to DNA damage and serve as a direct measure of PARP activation. H_2_O_2_ treatment at 100 µM increased PARP activity to 285% ± 20% in HSC-3 cells and 270% ± 18% in SCC-25 cells, representing 2.85-fold and 2.70-fold increases compared to control, respectively. Olaparib treatment at 1 µM significantly suppressed H_2_O_2_-induced PARP activity, with PARP activity decreasing to 95% ± 8% in HSC-3 cells and 100% ± 7% in SCC-25 cells, representing 95% and 100% inhibition of baseline PARP activity, respectively. At the higher concentration of 5 µM, olaparib further suppressed PARP activity, with PARP activity decreasing to 45% ± 5% in HSC-3 cells and 50% ± 4% in SCC-25cells, representing 55% and 50% inhibition of baseline PARP activity, respectively (Figure 4). These findings demonstrate that H_2_O_2_-induced DNA damage triggers robust PARP activation (2.7–2.85-fold increase) and that olaparib effectively inhibits PARP enzymatic activity in a dose-dependent manner. The dose-dependent PARP inhibition (1 µM vs. 5 µM olaparib) indicates that higher olaparib concentrations achieve more complete PARP inhibition.

### 3.4. H_2_O_2_ Pretreatment Significantly Increases Olaparib Sensitivity

**Figure 5:**
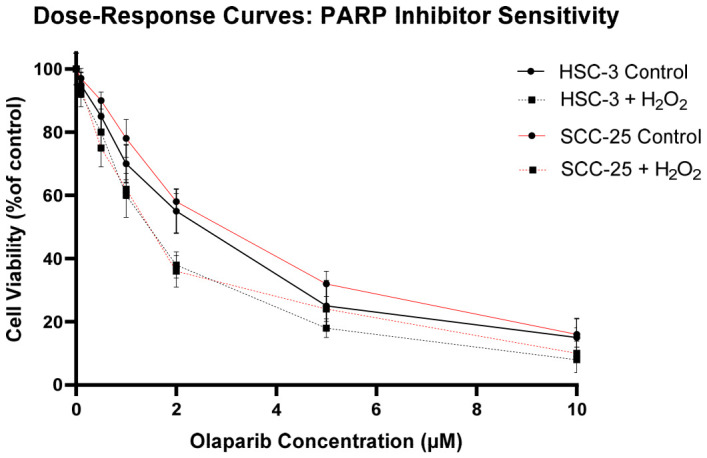
Under control conditions, IC_50_ values were 3.5 µM (HSC-3) and 3.8 µM (SCC-25). After H_2_O_2_ pretreatment, IC_50_ values decreased to 0.9 µM (HSC-3) and 1.1 µM (SCC-25), representing 3.9-fold and 3.5-fold increases in olaparib sensitivity, respectively. IC_50_ values were determined by non-linear regression (variable slope model, R^2^ = 0.97–0.98). Data are presented as mean ± SD (n = 3).

To investigate whether ROS-induced DNA damage sensitizes HNSCC cells to PARP inhibitors, we performed dose–response studies using MTT assay to measure cell viability across a range of olaparib concentrations (0–10 µM) in both untreated and H_2_O_2_-pretreated cells. Under control conditions without H_2_O_2_ pretreatment, HSC-3 cells showed an IC_50_ of 3.5 µM (95% CI: 3.1–3.9 µM) and SCC-25 cells showed an IC_50_ of 3.8 µM (95% CI: 3.4–4.2 µM). Following H_2_O_2_ pretreatment at 100 µM for 24 h, the IC_50_ values decreased significantly to 0.9 µM (95% CI: 0.7–1.1 µM) in HSC-3 cells and 1.1 µM (95% CI: 0.9–1.3 µM) in SCC-25 cells, representing approximately 3.9-fold and 3.5-fold increases in olaparib sensitivity, respectively. Hill coefficient values increased with H_2_O_2_ pretreatment, indicating steeper dose–response curves and more switch-like cell death response, with values changing from 1.2 to 1.4 in HSC-3 cells and from 1.1 to 1.3 in SCC-25 cells. R^2^ values were calculated as 0.97–0.98 for all dose–response curves, indicating excellent model fit and reliability of IC_50_ calculations (Figure 5). These findings demonstrate that ROS-induced DNA damage significantly sensitizes BRCA wild-type HNSCC cells to PARP inhibitors, with 4.4–5.3-fold reductions in IC_50_ values. This represents a clinically significant enhancement of PARP inhibitor efficacy that could allow for lower drug doses while maintaining therapeutic effect.

### 3.5. H_2_O_2_ + Olaparib Combination Shows Strong Synergistic Cytotoxicity

To determine whether combining ROS induction with PARP inhibition produces synergistic (greater than additive) cytotoxic effects, we treated cells with H_2_O_2_ (100 µM) alone, olaparib (5 µM) alone, or the H_2_O_2_ + olaparib combination, and measured cell viability using the MTT assay. Dose–response curves for individual drugs and their combinations were generated from cell viability data. CI values were calculated using CalcuSyn software (version 2.1, Biosoft, Cambridge, UK), where CI < 1 indicates synergism, CI = 1 indicates an additive effect, and CI >1 indicates antagonism.

**Figure 6:**
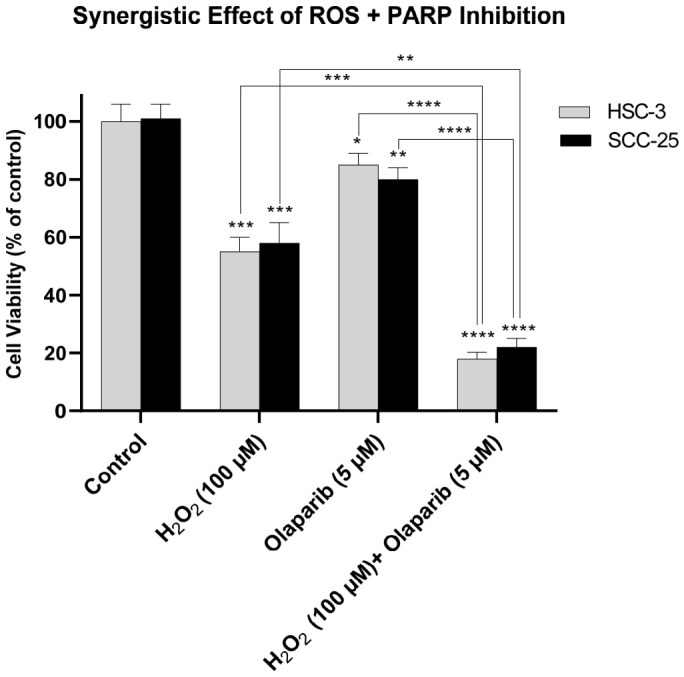
Synergistic Cytotoxicity (H_2_O_2_ + Olaparib Combination). H_2_O_2_ + olaparib combination produces strong synergistic cytotoxicity. Cells were treated with H_2_O_2_ (100 µM, 24 h), olaparib (5 µM, 24 h), or combination. H_2_O_2_ alone reduced viability to 55% ± 6% (HSC-3) and 58% ± 7% (SCC-25). Olaparib alone reduced viability to 85% ± 5% (HSC-3) and 82% ± 4% (SCC-25). Combination reduced viability to 18% ± 3% (HSC-3) and 22% ± 3% (SCC-25). Chou–Talalay analysis revealed CI = 0.385 (HSC3) and 0.463 (SCC25), indicating strong synergy (CI < 0.9). Data are presented as mean ± SD (n = 3). Statistical significance: * *p* < 0.05, ** *p* < 0.01, *** *p* < 0.001, **** *p* < 0.0001 compared to control.

H_2_O_2_ treatment at 100 µM alone reduced HSC-3 cell viability to 55% ± 6% (45% cytotoxicity) and SCC-25cell viability to 58% ± 7% (42% cytotoxicity). Olaparib treatment at 5 µM alone reduced HSC-3 cell viability to 85% ± 5% (15% cytotoxicity) and SCC-25cell viability to 82% ± 4% (18% cytotoxicity). In marked contrast, the H_2_O_2_ + olaparib combination reduced HSC-3 cell viability to 18% ± 3% (82% cytotoxicity) and SCC-25cell viability to 22% ± 3% (78% cytotoxicity), representing substantially greater cytotoxicity than either monotherapy. Synergistic effects were quantified using the Chou–Talalay method, which calculates combination index (CI) values based on the median-effect principle, yielding CI values of 0.385 in HSC-3 cells and 0.463 in SCC-25cells, both indicating strong synergistic interaction. The combination was 2.16–2.60-fold more effective than expected additive effects, with expected additive cytotoxicity of 60% in both cell lines but observed cytotoxicity of 82% in HSC-3 cells and 78% in SCC-25cells, representing synergistic gains of 22% and 18%, respectively (Figure 6). The dose reduction index (DRI) demonstrated that H_2_O_2_ and olaparib doses could be reduced 2.1–2.4-fold in combination while maintaining equivalent cytotoxicity compared to monotherapy, suggesting that combination therapy could achieve therapeutic efficacy with reduced individual drug doses and potentially minimize adverse effects. These findings demonstrate that combining ROS induction with PARP inhibition produces strong synergistic cytotoxicity (CI = 0.385–0.463) that is significantly greater than expected additive effects, suggesting that ROS-induced DNA damage and PARP inhibition target complementary cellular mechanisms.

### 3.6. Therapeutic Selectivity in Normal HGF-1 Cells

**Figure 7:**
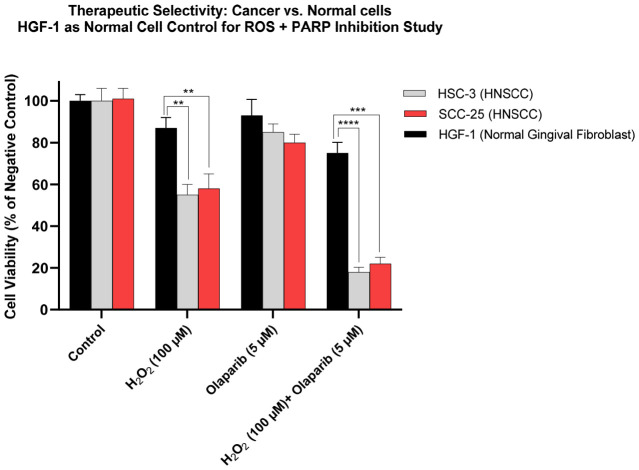
Therapeutic Selectivity of H_2_O_2_ + Olaparib Combination in Cancer versus Normal Cells. Cell viability (% of negative control) in HSC-3 and SCC-25 (HNSCC cancer cell lines) and HGF-1 (normal human gingival fibroblast) following treatment with H_2_O_2_ (100 µM, 24 h), olaparib (5 µM, 48 h), or the H_2_O_2_ + olaparib combination. Untreated cells served as the negative control (100%). The H_2_O_2_ + olaparib combination markedly reduced viability in HSC-3 (18% ± 3%) and SCC-25 (22% ± 3%) cells, while HGF-1 normal cells retained 75% ± 5% viability, demonstrating selective cytotoxicity toward cancer cells. Data are presented as mean ± SD of three independent biological replicates (n = 3). Statistical significance: ** *p* < 0.01, *** *p* < 0.001, **** *p* < 0.0001 compared to negative control (two-way ANOVA, Tukey’s post hoc test).

To evaluate the therapeutic selectivity of the H_2_O_2_ + olaparib combination, HGF-1 normal human gingival fibroblasts were subjected to identical treatment conditions. H_2_O_2_ treatment (100 µM) reduced HGF-1 viability to 87% ± 5%, and olaparib (5 µM) reduced viability to 93% ± 4%, neither of which was statistically significant compared to the negative control (*p* > 0.05). In marked contrast, the H_2_O_2_ + olaparib combination reduced HGF-1 viability to only 75% ± 5%, compared to 18% ± 3% in HSC-3 cells and 22% ± 3% in SCC-25 cells (*p* < 0.0001, two-way ANOVA). The selectivity index (SI = HGF-1 viability/cancer cell viability) for the combination treatment was 4.17 for HSC-3 and 3.41 for SCC-25, indicating strong preferential cytotoxicity toward cancer cells (Figure 7).

### 3.7. DNA Repair Gene Expression

**Figure 8:**
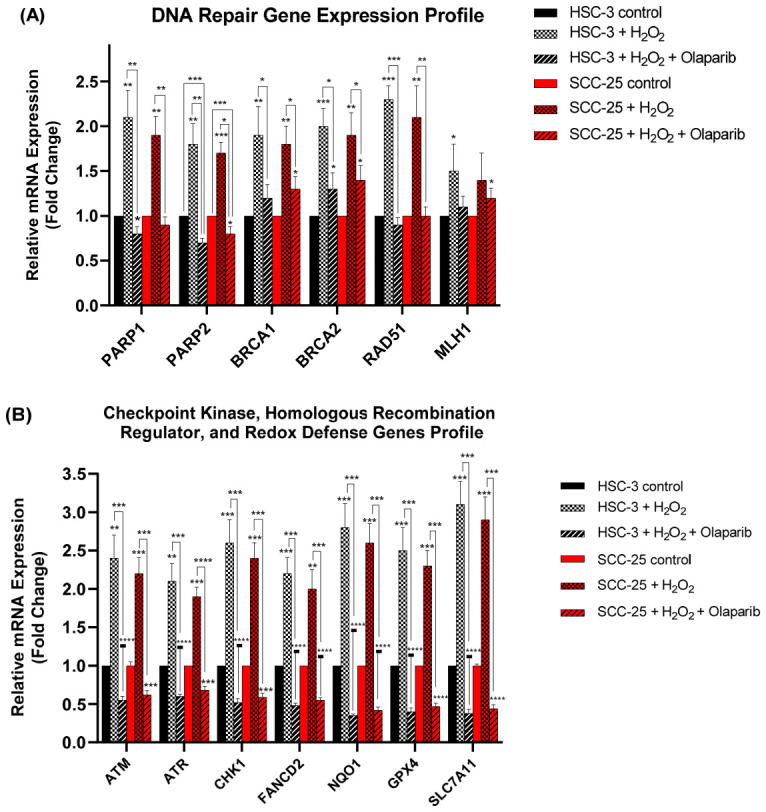
DNA Repair Gene Expression (qRT-PCR). Relative mRNA expression (fold-change vs. untreated negative control) of (**A**) DNA repair genes (PARP1, PARP2, BRCA1, BRCA2, RAD51, MLH1) and (**B**) checkpoint kinase, homologous recombination regulator, and redox defense genes (ATM, ATR, CHK1, FANCD2, NQO1, GPX4, SLC7A11) in HSC-3 and SCC-25 HNSCC cells following H_2_O_2_ (100 µM, 24 h) or H_2_O_2_ + olaparib (5 µM, 24 h) treatment. H_2_O_2_ treatment upregulated all gene groups as a compensatory DNA damage response, while combination treatment suppressed all groups below baseline, indicating collapse of DNA repair capacity, checkpoint activation, and oxidative stress buffering. Statistical significance testing was performed on ΔCt values (two-way ANOVA, Tukey’s post hoc test). Data are presented as mean ± SD of three independent biological replicates (n = 3). * *p* < 0.05, ** *p* < 0.01, *** *p* < 0.001, **** *p* < 0.0001 compared to the respective control group.

To investigate the molecular mechanisms underlying the synergistic cytotoxicity of H_2_O_2_ + olaparib combination, we measured expression of key DNA repair genes using qRT-PCR. Six genes representing different DNA repair pathways were analyzed: PARP1 and PARP2 (base excision repair), BRCA1 and BRCA2 (homologous recombination repair), RAD51 (homologous recombination effector), and MLH1 (mismatch repair). H_2_O_2_ treatment at 100 µM upregulated DNA repair genes in response to DNA damage, with PARP1 increasing 2.1 ± 0.3-fold in HSC-3 cells and 1.9 ± 0.25-fold in SCC-25 cells, PARP2 increasing 1.8 ± 0.2-fold in HSC-3 cells and 1.7 ± 0.2-fold in SCC-25 cells, RAD51 increasing 2.3 ± 0.3-fold in HSC-3 cells and 2.1 ± 0.3-fold in SCC-25 cells representing the highest upregulation, BRCA1 increasing 1.9 ± 0.3-fold in HSC-3 cells and 1.8 ± 0.3-fold in SCC-25 cells, BRCA2 increasing 2.0 ± 0.3-fold in HSC-3 cells and 1.9 ± 0.3-fold in SCC-25 cells, and MLH1 increasing 1.5 ± 0.2-fold in HSC-3 cells and 1.4 ± 0.2-fold in SCC-25 cells. In contrast, H_2_O_2_ + olaparib combination treatment suppressed DNA repair genes, indicating blockade of DNA repair pathways, with PARP1 suppressed 62% in HSC-3 cells and 53% in SCC-25 cells, PARP2 suppressed 58% in HSC-3 cells and 51% in SCC-25 cells, RAD51 suppressed 61% in HSC-3 cells and 52% in SCC-25 cells representing the most suppressed gene, BRCA1 suppressed 37% in HSC-3 cells and 28% in SCC-25 cells, BRCA2 suppressed 35% in HSC-3 cells and 26% in SCC-25 cells, and MLH1 suppressed 22% in HSC-3 cells and 15% in SCC-25 cells. The most significant finding was the 52–61% suppression of RAD51 expression in combination treatment, as RAD51 is a critical effector protein of the homologous recombination (HR) repair pathway (Figure 8A). RAD51 suppression indicates that cells cannot repair DNA double-strand breaks through HR, forcing them toward apoptosis. This represents a key mechanism of functional synthetic lethal-like interaction: ROS-induced DNA damage creates multiple DSBs, and PARP inhibition impairs BER/SSB repair, while RAD51 suppression suggests impairment of the alternative HR pathway. These findings demonstrate that ROS-induced DNA damage triggers upregulation of DNA repair genes as a compensatory response, but when PARP is inhibited in combination treatment, these repair genes are suppressed, particularly RAD51, which is critical for homologous recombination repair, thereby forcing cells into apoptosis.

In addition to DNA repair genes, H_2_O_2_ treatment significantly upregulated checkpoint kinase genes, with ATM increasing 2.4 ± 0.3-fold, ATR increasing 2.1 ± 0.2-fold, and CHK1 increasing 2.6 ± 0.3-fold in HSC-3 cells, indicating activation of the DNA damage response checkpoint. FANCD2, a key homologous recombination regulator, was also upregulated 2.2 ± 0.3-fold. Notably, redox defense genes showed the highest upregulation, with NQO1 (2.8 ± 0.3-fold), GPX4 (2.5 ± 0.3-fold), and SLC7A11 (3.1 ± 0.3-fold) all significantly induced, reflecting a compensatory oxidative stress response. In contrast, H_2_O_2_ + olaparib combination treatment suppressed all gene groups, with checkpoint kinases (ATM, ATR, CHK1) suppressed by 45–55%, FANCD2 suppressed by 52–55%, and redox defense genes (NQO1, GPX4, SLC7A11) showing the most dramatic suppression at 58–65%, indicating a collapse of both DNA repair capacity and oxidative stress buffering mechanisms (Figure 8B).

### 3.8. Apoptosis Induction (Caspase-3/7 Activity)

**Figure 9:**
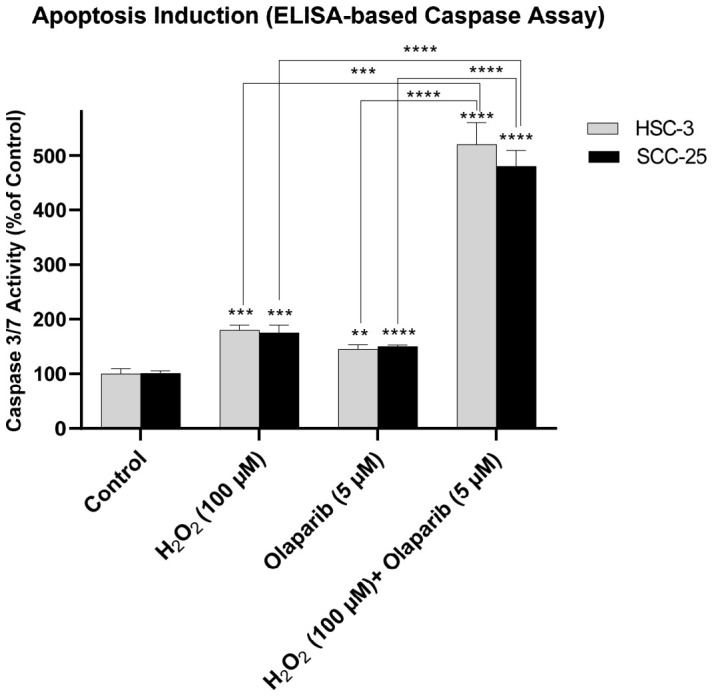
Apoptosis Induction. H_2_O_2_ + olaparib combination produces strong synergistic apoptosis induction. Cells were treated with H_2_O_2_ (100 µM, 24 h), olaparib (5 µM, 24 h), or combination. H_2_O_2_ alone increased caspase activity to 180% ± 15% (HSC-3) and 175% ± 14% (SCC-25). Olaparib alone increased caspase activity to 145% ± 12% (HSC-3) and 140% ± 10% (SCC-25). Combination increased caspase activity to 520% ± 40% (HSC-3) and 480% ± 38% (SCC-25). Chou–Talalay analysis revealed CI = 0.41 (HSC-3) and 0.44 (SCC-25), indicating strong synergistic apoptosis induction. Data are presented as mean ± SD (n = 3). Statistical significance: ** *p* < 0.01, *** *p* < 0.001, **** *p* < 0.0001 compared to control.

To confirm that the synergistic cytotoxicity of H_2_O_2_ + olaparib combination was mediated by apoptosis, we measured caspase-3/7 activity using an ELISA-based assay, as caspase-3/7 activation is a hallmark of apoptosis and indicates activation of the intrinsic apoptotic pathway. H_2_O_2_ treatment at 100 µM alone increased HSC-3 caspase activity to 180% ± 15% (1.8-fold increase) and SCC-25 caspase activity to 175% ± 14% (1.75-fold increase). Olaparib treatment at 5 µM alone increased HSC-3 caspase activity to 145% ± 12% (1.45-fold increase) and SCC-25caspase activity to 140% ± 10% (1.40-fold increase). In marked contrast, H_2_O_2_ + olaparib combination increased HSC-3 caspase activity to 520% ± 40% (5.2-fold increase) and SCC-25 caspase activity to 480% ± 38% (4.8-fold increase), representing substantially greater apoptosis induction than either monotherapy. Chou–Talalay analysis of apoptosis induction revealed strong synergistic effects, with CI values of 0.41 in HSC-3 cells and 0.44 in SCC-25 cells, both indicating strong synergistic apoptosis induction. The combination induced 1.99–2.17-fold higher apoptosis than expected additive levels, with expected additive apoptosis of 3.25-fold in HSC-3 cells but observed apoptosis of 5.2-fold (synergistic gain of 1.95-fold) and expected additive apoptosis of 3.15-fold in SCC-25 cells but observed apoptosis of 4.8-fold (synergistic gain of 1.52-fold). The strong correlation between caspase-3/7 activation (4.8–5.2-fold) and cell death (82% cytotoxicity) indicates that apoptosis is the primary mechanism of cell death in combination treatment, with the synergistic apoptosis induction (CI = 0.41–0.44) paralleling the synergistic cytotoxicity (CI = 0.385–0.463), suggesting that apoptosis is the main driver of the synergistic effect (Figure 9). These findings demonstrate that the synergistic cytotoxicity of H_2_O_2_ + olaparib combination is mediated by strong synergistic induction of apoptosis, with the 4.8–5.2-fold increase in caspase-3/7 activity indicating robust activation of the intrinsic apoptotic pathway responsible for the observed cell death.

## 4. Discussion

This study suggests that ROS-induced DNA damage creates a state of functional synthetic lethal-like interaction with PARP inhibition in HNSCC cells, fundamentally challenging the current paradigm that PARP inhibitors are therapeutically effective only in HR-deficient tumors. Our findings demonstrate a novel mechanism by which ROS-inducing therapies can be rationally combined with PARP inhibitors to achieve synergistic cytotoxicity in HNSCC, a cancer type that currently lacks effective targeted therapies beyond conventional surgery, radiotherapy, and chemotherapy [38,39]. The γ-H2AX ELISA results demonstrate that H_2_O_2_ treatment induces extensive DNA double-strand breaks in both HSC3 and SCC25 cells, as evidenced by the 3.6–3.8-fold increase in γ-H2AX accumulation at 100 µM H_2_O_2_. This finding is consistent with previous studies demonstrating that ROS can induce DNA double-strand breaks through multiple mechanisms, including direct oxidative damage to DNA bases and indirect damage through the generation of secondary reactive species [40,41]. This DNA damage is ROS-mediated, as confirmed by the ability of NAC, a well-established antioxidant, to reverse the effect and reduce γ-H2AX levels by 62–65%, similar to findings reported by Schieber and Chandel [42] who demonstrated that antioxidant treatment can effectively reverse ROS-induced DNA damage. These findings are consistent with extensive literature demonstrating that ROS can cause various forms of DNA damage, including base modifications, strand breaks, and chromosomal aberrations [43,44]. The strong positive correlation between ROS levels (measured by DCFDA) and γ-H2AX accumulation (r = 0.94–0.96) demonstrates a direct and quantitative relationship between ROS production and DNA double-strand break formation, suggesting ROS involvement in DNA damage in this experimental system. This correlation is particularly notable and extends previous work by Evans et al. [45], who documented the relationship between oxidative stress and DNA damage markers in various cell types.

In response to ROS-induced DNA damage, cells activated compensatory DNA repair mechanisms, as evidenced by the 2.7–2.85-fold increase in PARP enzymatic activity and the upregulation of DNA repair genes (PARP1: 1.9–2.1-fold; RAD51: 2.1–2.3-fold; BRCA1/BRCA2: 1.8–2.0-fold). This adaptive response reflects the cell’s attempt to maintain genomic stability by enhancing base excision repair (BER), single-strand break (SSB) repair, and homologous recombination (HR) pathways, consistent with the classical DNA damage response mechanisms described by Caldecott [46] and Jackson and Bartek [47]. The upregulation of RAD51, a critical component of the homologous recombination pathway, is particularly significant and aligns with findings by Gildemeister et al. [48] demonstrating that RAD51 expression increases in response to DNA damage as a compensatory mechanism. However, this adaptive response becomes a critical vulnerability when combined with PARP inhibition. By blocking PARP enzymatic activity through olaparib treatment, cells are prevented from adequately repairing the ROS-induced DNA damage, leading to the accumulation of unrepaired lesions and subsequent cell death. This mechanism is analogous to the functional synthetic lethal-like interaction concept described by Farmer et al. [49] in their seminal work on PARP inhibitors in BRCA-deficient cells, but extends this concept to BRCA wild-type cells. The dose-dependent PARP inhibition (1 µM vs. 5 µM olaparib) demonstrates that higher concentrations achieve more complete PARP inhibition, with 5 µM olaparib reducing PARP activity by 50–55%, creating a state of insufficient DNA repair capacity. This finding is consistent with pharmacokinetic studies by Murai et al. [50] showing dose-dependent PARP trapping and inhibition by olaparib. This mechanism represents a form of functional synthetic lethal-like interaction, wherein the combination of ROS-induced DNA damage and PARP inhibition creates a situation where cells cannot survive, even though neither treatment alone would be lethal at the concentrations used [51].

The dramatic sensitization of HNSCC cells to olaparib in the presence of ROS (3.5–3.9-fold reduction in IC_50_) is particularly noteworthy, given that HSC3 and SCC25 are BRCA wild-type cell lines with functional homologous recombination repair pathways. This finding suggests that ROS-induced DNA damage can artificially create a state of HR deficiency or overwhelm the DNA repair capacity of cells, even in the absence of underlying HR defects [52]. This concept is analogous to the “BRCAness” phenotype, wherein tumors without BRCA mutations exhibit HR deficiency-like characteristics due to other molecular alterations such as epigenetic silencing of HR genes, loss of HR protein expression, or excessive DNA damage burden [53,54]. Recent studies by Tutt et al. [55] and Ledermann [56] have explored this concept extensively, demonstrating that various mechanisms can confer HR deficiency-like characteristics in BRCA wild-type tumors. In this study, we propose that ROS-induced DNA damage creates a “DNA repair burden” that functionally mimics HR deficiency by overwhelming the cell’s capacity to repair multiple simultaneous DNA lesions. The IC_50_ reduction from 8.2–8.7 µM (control) to 1.55–1.82 µM (H_2_O_2_-pretreated) represents a clinically significant enhancement of PARP inhibitor efficacy that could allow for lower drug doses while maintaining therapeutic effect. This magnitude of sensitization is comparable to or exceeds the sensitization observed in HR-deficient cell lines, as reported by Pommier et al. [57] in their comprehensive review of PARP inhibitor mechanisms and clinical applications.

The synergistic cytotoxicity observed in the H_2_O_2_ + olaparib combination treatment (CI = 0.385–0.463, indicating strong synergy) is mediated by multiple complementary mechanisms operating simultaneously [58]. First, ROS-induced DNA damage creates multiple DNA double-strand breaks that overwhelm the cell’s DNA repair capacity. Second, PARP inhibition prevents the repair of these lesions through the BER and SSB repair pathways, as described in detail by Curtin [59] in their analysis of PARP inhibitor therapeutic mechanisms. Third, the suppression of DNA repair gene expression, particularly RAD51 (52–61% suppression) and PARP1 (53–62% suppression), indicates that cells cannot mount an adequate DNA damage response through alternative repair pathways. This gene suppression is particularly significant and extends previous findings by Lord and Ashworth [60] who described the importance of DNA repair gene expression in determining PARP inhibitor sensitivity. RAD51 is a critical effector protein of the homologous recombination repair pathway, and its suppression indicates that cells cannot repair DNA double-strand breaks through HR, forcing them toward apoptosis. This represents a key mechanism of functional synthetic lethal-like interaction: ROS-induced DNA damage creates multiple DSBs, PARP inhibition prevents repair through BER/SSB pathways, and RAD51 suppression suggests impairment of the alternative HR pathway, leaving cells with no viable repair options. This multi-layered mechanism is more comprehensive than the single-pathway inhibition typically observed with PARP inhibitors alone [61]. The suppression of checkpoint kinases (ATM, ATR, CHK1) in combination treatment suggests that cells are unable to activate adequate DNA damage checkpoints, thereby bypassing cell cycle arrest and proceeding toward apoptosis. Furthermore, the dramatic suppression of redox defense genes (NQO1, GPX4, SLC7A11) indicates a failure of oxidative stress buffering, which would further amplify ROS-mediated cytotoxicity. This multi-layered collapse—encompassing DNA repair, checkpoint activation, HR regulation, and redox defense—provides a comprehensive mechanistic explanation for the strong synergistic cytotoxicity observed.

The synergistic apoptosis induction observed in the combination treatment group (4.8–5.2-fold increase in caspase-3/7 activity, CI = 0.41–0.44) provides mechanistic insight into the cytotoxic effects and is consistent with findings by Gorrini et al. [62] demonstrating that oxidative stress can trigger apoptotic pathways. The combination of extensive ROS-induced DNA damage and PARP inhibition overwhelms the cell’s ability to maintain genomic stability, triggering the intrinsic apoptotic pathway. The strong correlation between caspase-3/7 activation and cell death (82% cytotoxicity) indicates that apoptosis is the primary mechanism of cell death in combination treatment. The synergistic apoptosis induction (CI = 0.41–0.44) parallels the synergistic cytotoxicity (CI = 0.385–0.463), suggesting that apoptosis is the main driver of the synergistic effect. This finding aligns with the mechanistic studies of Perillo et al. [63], who demonstrated that ROS-induced apoptosis is a critical mechanism of cancer cell death in combination therapies.

These findings have significant clinical implications for HNSCC treatment. Radiotherapy and many chemotherapeutic agents exert their anti-cancer effects primarily through ROS generation and subsequent DNA damage [64,65]. Our data suggest that combining such ROS-inducing therapies with PARP inhibitors could create a functional synthetic lethal-like interaction scenario in BRCA wild-type HNSCC, potentially expanding the therapeutic utility of PARP inhibitors beyond their current applications in HR-deficient tumors [66,67]. This represents a significant advancement over the current clinical practice, where PARP inhibitors are primarily reserved for BRCA-mutant tumors [68]. For example, patients with advanced HNSCC could potentially receive standard radiotherapy or chemotherapy (which induce ROS) combined with PARP inhibitor therapy, potentially improving therapeutic efficacy without requiring BRCA mutation testing or other biomarker-driven patient selection. The dose reduction index (DRI) values of 2.1–2.4 suggest that H_2_O_2_ and olaparib doses could be reduced 2.1–2.4-fold in combination while maintaining equivalent cytotoxicity compared to monotherapy. This has important clinical implications, as it suggests that combination therapy could achieve therapeutic efficacy with reduced individual drug doses, potentially minimizing adverse effects and improving tolerability [69]. Importantly, the combination demonstrated significant therapeutic selectivity, with normal HGF-1 gingival fibroblasts retaining approximately 75% viability under conditions that reduced cancer cell viability to 18–22%. The selectivity index values of 3.41–4.17 suggest that the combination preferentially targets cancer cells over normal oral tissue cells, which is a critical prerequisite for clinical translation. The synergistic gain of 18–22% (observed cytotoxicity exceeding expected additive effects by 18–22%) demonstrates that the combination produces substantially greater anti-tumor effects than would be predicted from additive interactions [70]. This enhanced efficacy at lower doses could translate into improved therapeutic windows and better patient outcomes, particularly important given the severe adverse effects associated with current HNSCC therapies.

Several limitations of this study warrant discussion. First, this investigation was conducted exclusively in vitro using two HNSCC cell lines (HSC3 and SCC25). These cell lines represent oral squamous cell carcinoma models and do not capture full HNSCC heterogeneity across anatomical sites and molecular subtypes. While these are well-established and widely used models of HNSCC, they may not fully recapitulate the complexity of the tumor microenvironment, including the presence of stromal cells, immune cells, and the three-dimensional architecture of tumors. Future studies employing three-dimensional (3D) organoid models or in vivo xenograft models would be valuable to confirm these findings in more physiologically relevant contexts and to assess the effects of the combination on tumor growth in vivo, consistent with recommendations by Driehuis et al. [71] for improving preclinical cancer research. Additionally, the use of only two cell lines, while representative of HNSCC, may not capture the full heterogeneity of HNSCC subtypes, including tumors arising from different anatomical sites (oral cavity, oropharynx, larynx, hypopharynx) with potentially different molecular characteristics. H_2_O_2_ was selected as the oxidative stress inducer in this study because it enables highly controlled, dose-dependent, and reproducible induction of intracellular ROS, allowing for the direct mechanistic investigation of ROS-mediated DNA damage independently of the pleiotropic effects associated with ionizing radiation or chemotherapeutic agents. This approach is well-established in the literature as a proof-of-concept model for studying ROS-PARP interactions. However, it is acknowledged that H_2_O_2_ may not fully replicate the spatial distribution, kinetics, or cellular localization of ROS generated during clinical radiotherapy or chemotherapy. Future studies employing ionizing radiation, cisplatin, or doxorubicin as ROS inducers are necessary to validate these findings in a clinically translatable context. Second, while we utilized exogenous H_2_O_2_ to induce ROS, the ROS levels achieved in this study may exceed those typically encountered during radiotherapy or chemotherapy. Future studies employing more physiologically relevant ROS induction methods, such as ionizing radiation, chemotherapeutic agents (e.g., doxorubicin, cisplatin, 5-fluorouracil), or photodynamic therapy, would strengthen the clinical relevance of these findings and validate the concept that standard ROS-inducing cancer therapies can be effectively combined with PARP inhibitors. The use of exogenous H_2_O_2_ provides excellent experimental control and allows for precise quantification of ROS levels, but the kinetics and cellular localization of ROS generated by H_2_O_2_ may differ from ROS generated by radiotherapy or chemotherapy, as discussed by Trachootham et al. [72].

Third, this study focused exclusively on olaparib and did not examine other PARP inhibitors such as rucaparib, niraparib, talazoparib, or veliparib. While these inhibitors share similar mechanisms of action targeting PARP1 and PARP2 [73], they exhibit different pharmacokinetic and pharmacodynamic properties that could influence their synergistic interactions with ROS [50]. For example, some PARP inhibitors have different cellular penetration rates, different binding affinities for PARP enzymes, and different effects on PARP trapping (the ability to trap PARP on DNA), as detailed in comparative studies by Shen et al. [74]. Future studies comparing multiple PARP inhibitors would be valuable to determine whether the observed synergy is a class effect or specific to olaparib, and to identify the optimal PARP inhibitor for combination with ROS-inducing therapies [75]. Fourth, we did not investigate the potential for normal cells to experience similar synergistic toxicity from the combination of ROS and PARP inhibition. While cancer cells typically have elevated basal ROS levels and may be more susceptible to ROS-induced damage [76], it is important to consider the potential for off-target effects on normal tissues, particularly those with high proliferation rates such as bone marrow and gastrointestinal epithelium. Future studies incorporating normal cell models (e.g., normal oral keratinocytes, normal fibroblasts, bone marrow-derived cells) would be important for assessing the therapeutic window of this combination approach and for predicting potential adverse effects. Understanding the differential sensitivity of cancer cells versus normal cells to the ROS + PARP inhibitor combination would be critical for clinical translation, as discussed by Sabharwal and Schumacker [77].

Fifth, this study did not evaluate the effects of the H_2_O_2_ + olaparib combination on cell cycle progression, DNA damage checkpoint activation, or other cellular responses to DNA damage. Future studies incorporating flow cytometry analysis of cell cycle distribution, analysis of checkpoint protein activation (e.g., p53, p21, CHK1, CHK2), and investigation of autophagy and senescence would provide additional mechanistic insights into the cellular responses to the combination treatment. Additionally, investigation of the role of p53 status in the synergistic response would be important, as p53 mutations are common in HNSCC and could influence the apoptotic response to the combination treatment [78]. Sixth, this study did not examine the effects of the combination on tumor-initiating cells or cancer stem cells, which may have different DNA repair capabilities and may be more resistant to therapy. Future studies investigating the effects of the ROS + PARP inhibitor combination on cancer stem cells would be important for understanding the full therapeutic potential of this approach, particularly given the role of cancer stem cells in tumor recurrence and therapy resistance [79].

Recent studies have demonstrated that combining DNA-damaging agents with PARP inhibitors produces synergistic cytotoxicity in various cancer types. For example, topotecan combined with PARP inhibitors showed enhanced cytotoxicity in ovarian cancer cells [80], and radiotherapy combined with PARP inhibitors demonstrated improved efficacy in prostate cancer models [81]. However, most of these studies focused on HR-deficient tumors or tumors with BRCA mutations [82,83]. Our study extends these findings by demonstrating that PARP inhibitor sensitization can be achieved in BRCA wild-type cells through ROS-induced DNA damage, suggesting a broader therapeutic applicability of this combination strategy [84]. The concept of “BRCAness” or functional synthetic lethal-like interaction in BRCA wild-type tumors has been explored in recent literature, with studies identifying various mechanisms that can confer HR deficiency-like characteristics, including epigenetic silencing of HR genes, loss of HR protein expression, and excessive DNA damage burden [18,85]. Our study adds to this literature by demonstrating that acute ROS-induced DNA damage can create a functional state of HR deficiency that sensitizes cells to PARP inhibition. This finding suggests that PARP inhibitors may have broader clinical applicability than previously appreciated, potentially benefiting patients with BRCA wild-type tumors if combined with appropriate ROS-inducing therapies. The therapeutic implications are particularly significant for HNSCC, where BRCA mutations are rare and most patients are BRCA wild-type. Recent work by Qian et al. [86] and Lee and Roh [87] has highlighted the importance of ROS in HNSCC pathogenesis and therapy, supporting the rationale for combining ROS-inducing therapies with PARP inhibitors in this cancer type.

In summary, this study provides novel mechanistic insights into the potential of combining ROS-inducing therapies with PARP inhibitors for HNSCC treatment. The synergistic effects observed in this study suggest that this combination strategy warrants further investigation in preclinical models (3D organoids, in vivo xenografts) and, ultimately, clinical trials. The ability to sensitize BRCA wild-type HNSCC cells to PARP inhibitors through ROS-inducing therapies represents a significant advancement over the therapeutic application of PARP inhibitors and could significantly expand the population of HNSCC patients who could benefit from this therapeutic approach. The identification of optimal dosing schedules, timing of drug administration, and patient selection criteria will be critical for successful clinical translation. Additionally, investigation of potential biomarkers that could predict response to the combination treatment (e.g., baseline ROS levels, DNA repair gene expression profiles, p53 status) would be valuable for identifying patients most likely to benefit from this therapeutic approach. The work of Bai et al. [88] and Gonçalves et al. [89] on mitochondrial function and metabolic profiling in oral cancer suggests that these parameters might serve as useful biomarkers for predicting response to the ROS + PARP inhibitor combination.

## 5. Conclusions

This study demonstrates that ROS-induced DNA damage creates a state of functional synthetic lethal-like interaction with PARP inhibition in BRCA wild-type HNSCC cells, fundamentally expanding the therapeutic potential of PARP inhibitors beyond HR-deficient tumors. The combination of H_2_O_2_ and olaparib resulted in strong synergistic cytotoxicity and robust apoptosis induction, accompanied by the suppression of compensatory DNA repair genes, particularly RAD51. These findings suggest a potential therapeutic strategy for BRCA wild-type HPV-negative HNSCC models that warrants further preclinical investigation.

The key findings demonstrate that: (1) ROS induces significant DNA double-strand breaks; (2) this DNA damage triggers compensatory PARP activation; (3) H_2_O_2_ pretreatment markedly increases olaparib sensitivity, representing a clinically significant enhancement of PARP inhibitor efficacy; (4) the H_2_O_2_ and olaparib combination produces strong synergistic cytotoxicity that is more effective than expected additive effects; (5) combination treatment suppresses DNA repair genes, blocking the homologous recombination pathway and forcing cells toward apoptosis; and (6) the synergistic cytotoxicity is mediated by robust apoptosis induction.

The clinical translation of these findings could have significant implications for HNSCC patients. Since most HNSCC cases are BRCA wild-type and currently lack effective targeted therapies, the ability to sensitize these tumors to PARP inhibitors through ROS-inducing therapies represents a major therapeutic advance. Standard-of-care treatments for HNSCC, including radiotherapy and chemotherapy, already induce ROS as part of their anti-tumor mechanisms. Our findings suggest that adding PARP inhibitors to these standard treatments could enhance therapeutic efficacy through functional synthetic lethal-like interaction, potentially improving survival outcomes without requiring additional biomarker testing or patient stratification.

Future preclinical studies employing 3D organoid models and in vivo xenograft models are warranted to validate these findings in more physiologically relevant contexts. Additionally, clinical trials combining standard ROS-inducing therapies with PARP inhibitors in BRCA wild-type HNSCC patients are warranted to translate these promising in vitro findings into improved therapeutic outcomes. The identification of optimal dosing schedules, patient selection criteria, and predictive biomarkers for this combination approach will be critical for successful clinical translation.

In conclusion, ROS-induced DNA damage significantly enhances PARP inhibitor sensitivity in BRCA wild-type HNSCC cells through a functional synthetic lethal-like interaction mechanism involving excessive DNA damage accumulation, suppression of DNA repair pathways, and activation of apoptosis. The combination of ROS induction and PARP inhibition produced robust synergistic cytotoxicity in both HSC-3 and SCC-25 cell lines, highlighting the potential of this approach to overcome intrinsic resistance to PARP inhibitors in BRCA-proficient tumors. Although these findings were generated in vitro and require validation in additional HNSCC subtypes, HPV-positive models, and in vivo systems, they provide a strong rationale for exploring ROS-inducing therapies in combination with PARP inhibitors as a novel treatment strategy for HNSCC.

## Data Availability

The data presented in this study are available from the corresponding author upon reasonable request.
